# Interplay of n-3 Polyunsaturated Fatty Acids, Intestinal Inflammation, and Gut Microbiota in Celiac Disease Pathogenesis

**DOI:** 10.3390/nu17040621

**Published:** 2025-02-09

**Authors:** Karla A. Bascuñán, Magdalena Araya, Juan Manuel Rodríguez, Leda Roncoroni, Luca Elli, Josefina Del Pilar López Alvarez, Rodrigo Valenzuela

**Affiliations:** 1 Department of Nutrition, Faculty of Medicine, University of Chile, Santiago 8380453, Chile; kbascunan@uchile.cl (K.A.B.); josefinalopez@ug.uchile.cl (J.D.P.L.A.); 2 Institute of Nutrition and Food Technology (INTA), University of Chile, Santiago 7830490, Chilejuan.rodriguez@inta.uchile.cl (J.M.R.); 3 Center for Prevention and Diagnosis of Celiac Disease, Gastroenterology and Endoscopy Unit, Fondazione IRCCS Ca’ Granda Ospedale Maggiore Policlinico, 20122 Milan, Italy; leda.roncoroni@policlinico.mi.it (L.R.); luca.elli@policlinico.mi.it (L.E.); 4 Department of Biomedical, Surgical and Dental Sciences, University of Milan, 20122 Milan, Italy; 5 Department of Pathophysiology and Transplantation, Università degli Studi di Milano, 20122 Milan, Italy

**Keywords:** celiac disease, fatty acids, inflammation, microbiota, gluten, intestine

## Abstract

Celiac disease (CD) is a chronic autoimmune disorder driven by both genetic and environmental factors, with the HLA DQ2/DQ8 genotypes playing a central role in its development. Despite the genetic predisposition, only a small percentage of individuals carrying these genotypes develop the disease. Gluten, a protein found in wheat, rye, and barley, is the primary environmental trigger, but other factors, such as the intestinal microbiota, may also contribute to disease progression. While the gluten-free diet (GFD) remains the cornerstone of treatment, many CD patients experience persistent inflammation and gut dysbiosis, leading to ongoing symptoms and complications. This chronic inflammation, which impairs nutrient absorption, increases the risk of malnutrition, anemia, and other autoimmune disorders. Recent studies have identified an altered gut microbiota in CD patients, both on and off the GFD, highlighting the potential role of the microbiota in disease pathogenesis. An emerging area of interest is the supplementation of n-3 polyunsaturated fatty acids (PUFAs), known for their anti-inflammatory properties, as a potential therapeutic strategy. n-3 PUFAs, found in fish oil and certain plant oils, modulate the immune cell function and cytokine production, making them a promising intervention for controlling chronic inflammation in CD. This review explores the current understanding of n-3 PUFAs’ effects on the gut microbiota’s composition and inflammation in CD, with the goal of identifying new avenues for complementary treatments to improve disease management.

## 1. Introduction

Celiac disease (CD) is a chronic autoimmune condition with a strong genetic component. It is characterized by multi-organ inflammatory phenomena that lead to intestinal damage, precipitated by dietary gluten in genetically susceptible individuals [1,2]. Globally, CD affects approximately 1% of the population [3]. The incidence of CD is present in all populations where it has been sought and has consistently increased in recent decades worldwide [4] with an annual average increase of 7.5% [5] (for further information see references [2,3,5]). Serological markers for CD autoimmunity have improved its diagnosis, and their presence strongly suggests histological damage in small intestinal biopsies; it is this latter presence that confirms the diagnosis. The economic costs associated with CD are substantial for patients and healthcare services [6], and the quality of life is frequently compromised [7] with several factors influencing the phenomenon [8]. Recent studies show that the quality of life and adherence are related, i.e., the less adherence to treatment (i.e., gluten-free diet) the more damaged the quality of life is [9].

The development of CD involves a combination of genetic and environmental factors. Genetics, particularly the human leukocyte antigen (HLA) DQ2/DQ8 genotypes, play a significant role in CD development [10]. However, it is important to note that while 30–40% of people carry these genotypes, only 1% develop the disease [11]. The HLA locus accounts for about 25–40% of the genetic variance in CD [12,13,14] and genome-wide association studies have only explained about 5–10% of the disease risk, leaving much of the risk unexplained [15]. The second major factor is gluten, which refers to certain proteins of wheat, rye, and barley [2]. Despite the significance of these factors, there is still a limited understanding of many aspects of the inflammation present in the intestine and the impact of other factors, for example, the intestinal microbiota. A gluten-free diet (GFD) is the main treatment for CD, providing the relief of symptoms and promoting the healing of the intestinal lining in most cases [16]. However, the improvement in adult patients may be slow and sometimes inconsistent, even when strictly following a GFD [17,18].

Intestinal inflammation in CD is driven by an autoimmune response to gluten, leading to the damage of the intestinal epithelium and villous atrophy [19]. This immune response involves the activation of different immune cells, causing chronic inflammation that impairs nutrient absorption. Even with a GFD, many CD patients experience persistent low-grade inflammation and gut dysbiosis, which can contribute to ongoing symptoms and complications [20]. This chronic inflammation increases the risk of malnutrition, anemia, and other autoimmune diseases [21]. The challenge in managing CD lies in effectively controlling this inflammation and restoring the intestinal health, emphasizing the need for additional therapeutic approaches beyond solely a gluten-free diet [22].

Intestinal dysbiosis has been observed in patients with CD, both untreated and on a GFD. Several studies have identified distinct bacterial populations associated with CD and healthy individuals [23]. A key challenge that deserves attention is the role of n-3 polyunsaturated fatty acids (n-3 PUFAs). They may alter dysbiosis, and this could influence the course of the disease. Investigating the potential effects of n-3 PUFAs and their role in modulating the inflammation in CD could provide valuable insights that could lead to new therapeutic approaches for managing the disease [24].

PUFAs, including omega-3 (n-3) and omega-6 (n-6) fatty acids, are relevant lipids that significantly influence the inflammatory processes. n-3 PUFAs, particularly found in fish oil and certain plant oils, are known for their anti-inflammatory properties, which involve the modulation of cytokine production and eicosanoid synthesis [25]. These fatty acids play a critical role in autoimmune diseases by affecting the immune cell function and cytokine profiles. In this regard, n-3 PUFAs have been shown to regulate the activity of T cells, macrophages, and other immune cells, thereby reducing the excessive inflammation [26]. It has been postulated that n-3 PUFAs may be a promising therapeutic approach for managing diseases characterized by chronic inflammation, such as rheumatoid arthritis, inflammatory bowel disease, and celiac disease [27]. To evaluate the potential impact of n-3 PUFA supplementation, we here conduct a comprehensive review of the literature on their role in modulating the intestinal inflammation and dysbiosis in CD.

## 2. Methodology

The literature search was conducted across Medline (Pubmed), Scopus, the Web of Science, CINAHL, and Cochrane databases, focusing on human studies published between January 2010 and September 2023. The search strategy involved combinations of terms such as “omega-3 AND Celiac disease”, “fatty acids AND inflammation”, “microbiota AND PUFA”, “PUFA supplementation AND intestine”, and “omega-3 OR n-3 OR PUFA AND inflammation AND intestine”. Observational, case-control, retrospective, and randomized controlled studies published in English were included. Additionally, relevant narrative reviews in English were incorporated to provide context and a broader perspective on the effects of omega-3 and PUFA on celiac disease, inflammation, and gut health.

## 3. From the Celiac Immune Response to Inflammation

The adaptive immune response is a well-established CD effector system. It begins with the deamidation of gliadin peptides (gluten peptides derived from wheat digestion) by tissue transglutaminase 2 (TTG), the main autoantigen in CD [28]. Deamidation promotes the formation of a complex with a strong affinity for HLA-DQ2/DQ8 molecules in antigen-presenting cells [2], making it easier to present it to gliadin-reactive CD4+ T cells [29], and leading to the activation of the local inflammatory cascade. Throughout this process, antibodies to TTG, gliadin, and actin are produced by unclear mechanisms. TTG antibodies measured in the blood are currently the most sensitive and specific way to screen for CD.

**Figure 1:**
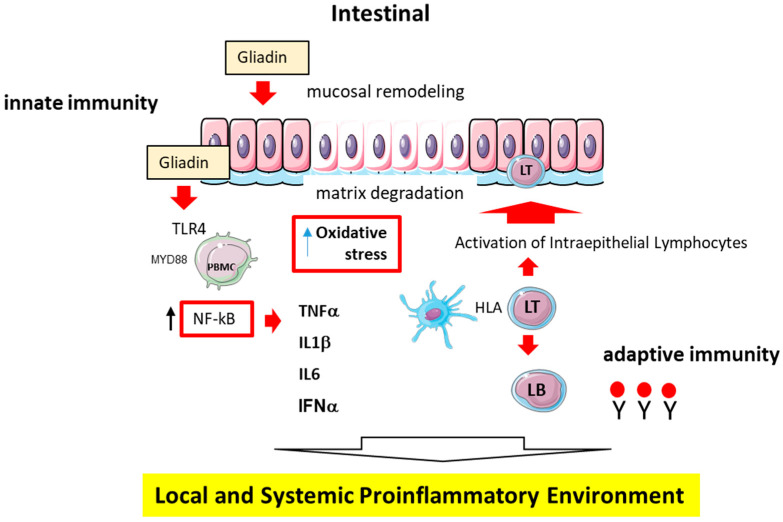
Interaction between innate and adaptive immunity in celiac disease. The inflammatory process underlying celiac disease involves the coordinated activation of both innate and adaptive immunity, which together contribute to a pro-inflammatory microenvironment at both local and systemic levels. Immune cell-derived cytokines in response to gluten disrupt the intestinal epithelium, driving the pathological features of celiac disease. This immune response includes the activation of dendritic cells and macrophages (innate immunity), as well as the subsequent recruitment and activation of T cells (adaptive immunity), which perpetuate epithelial damage and the chronic inflammatory state.

The role of CD4 T cells in initiating and organizing the adaptive immune response in CD is well supported by evidence [30,31]. These cells can modify the intestinal inflammatory response by producing numerous cytokines [32]. Gliadin-specific CD4+ T lymphocytes from the mucosa of CD patients mainly produce interferon (IFN)-γ and are primarily of the Th1 type [32]. The Th17 response is also relevant in this process [33]. When activated, CD4 T cells initiate mucosal damage, which is the basis for diagnosis through an intestinal biopsy [34]. The persistent release of pro-inflammatory cytokines, including INF-γ, maintains the pro-inflammatory environment, leading to mucosal deterioration [35]. Additionally, metalloproteinase activation leads to characteristic architectural changes in the mucosa with villus flattening [36]. It is now widely accepted that there is a pro-inflammatory environment in the small intestinal mucosa in CD [37] (Figure 1).

While adaptive immunity was historically thought to be the only immune mechanism involved, it is now known that the innate immune system also participates by promoting TTG-mediated gliadin deamidation. This recent recognition is significant [37] because it was believed that the innate response was primarily targeted against relatively uniform microbial patterns of antigens (pathogen-associated molecular patterns, PAMPs). A current hypothesis suggests that the innate response can also be triggered by proteins found in “cereal” constituents [38,39]. Gliadin peptides can directly initiate innate immune responses in macrophages and dendritic cells through pattern recognition receptors (such as Toll-like receptor 4 and MyD88-dependent pathways), which promote NF-κB translocation, leading to the production of interleukin (IL) IL-1β, IL6, and tumor necrosis factor α (TNF-α) [40], and these, in turn, can enhance the adaptive immune response [28]. Although several mechanisms of action for innate and adaptive immunity contributing to CD have been described, it is still unclear how non-gluten environmental factors trigger CD4 cell activation and its subsequent maintenance.

The inflammatory process in CD is also related to oxidative stress (OS). Reactive oxygen species (ROS) are highly reactive and dangerous molecules constantly produced in cells [41]. Normally, the body has natural defense mechanisms, such as superoxide dismutase, glutathione peroxidase/reductase, and antioxidants like glutathione and vitamins to counteract the harmful effects of ROS [42,43]. However, when the production of ROS exceeds the body’s antioxidant capacity, OS occurs [44]. The OS can damage cell proteins, DNA, and lipids, and is implicated in the development of many human diseases, including CD [45]. ROS can trigger the production of transcription factors that regulate the synthesis of antioxidant proteins and pro-inflammatory factors, leading to increased inflammation and the activation of immune cells [46]. The molecular mechanisms involved in CD are still not fully understood [47]. In the past, studies were limited to small intestine biopsies, but now, less invasive methods like the assessment of peripheral blood mononuclear cells (PBMC) are available. This method has revealed a significant dysfunction in the glutathione redox cycle and reduced its ability to regenerate glutathione and detoxify lipid hydroperoxide (LOOH) [47]. Several studies have suggested that gluten may lead to an imbalance in oxidative processes in celiac patients, resulting in higher levels of lipid peroxidation products and changes in the oxidized/reduced glutathione ratio [48]. An emerging approach to reduce ROS in autoimmune diseases involves regulating the balance of n-3/n-6 PUFAs, although evidence specific to CD is lacking [49].

## 4. Gluten, the Intestinal Microbiota, and the Immune Response

There is a close relationship between gluten metabolism, the gut microbiota, and the pathophysiology of CD. Alterations in small intestinal microbial composition have recently been associated with active CD, indicating a possible role for the microbiota in this condition [50]; interrelations of microbiota communities and the mucosa define the intestinal health and homeostasis. By modifying the intestinal microbiota, one can modulate the mucosal immune responses [51]. The current evidence indicates that dysbiosis [52], compositional and functional gut microbiome alterations, is present in CD [23], particularly affecting beneficial bacteria. CD patients on a GFD have significantly reduced *Lactobacillus* and *Bifidobacterium* diversity [53]. Although the studies are still insufficient, a dysbiotic microbiota seems to be associated with persistent gastrointestinal symptoms in treated CD patients, suggesting a pathogenic role in these cases [54]. Given that the microbiota is modifiable through the diet [55], one may postulate that the presence/absence of gluten could change the diversity and proportions of the microbial communities constituting the gut microbiota [23]. Microbial metabolism produces short-chain fatty acids (SCFAs), including butyrate, acetate, and propionate, which profoundly influence the gastrointestinal tract physiology [56]. An interesting question is whether GFD plus a lipid-modified diet (i.e., with increased n-3 PUFAs) could modulate the inflammatory processes and intestinal health. On the other hand, a final multivariate approach combining both viable counts and metabolites suggested that gluten-free bread could beneficially modulate the celiac gut microbiome. Although interesting, unfortunately, these ideas remain to be proved, and there are no human studies to support them [57]. Additionally, the consumption of pure oats within a gluten-free diet does not induce dysbiosis in the fecal microbiota of individuals with celiac disease or non-celiac gluten sensitivity, despite the diet being associated with higher fat intake and lower fiber intake. These findings indicate that pure oats may be a promising addition to gluten-free diets, particularly for individuals with minor microbiota disorders [58].

## 5. n-6 and n-3 PUFA Nutritional and Metabolic Aspects

The n-6 and n-3 PUFAs have diverse and relevant functions in organisms, particularly in tissue growth and development [59,60] in addition to robust evidence demonstrating the importance of these FA in the regulation of vascular homeostasis and the inflammatory response [61]. In this regard, linoleic acid (C18:2n-6) and α-linolenic acid (C18:3n-3, ALA) are essential FAs and precursors of other PUFAs. Both FAs in mammals can be converted into other PUFAs mainly in the liver, through a complex process of desaturation and elongation that involves desaturases (Δ-5D and Δ-6D) that insert double bonds and elongases that introduce two-carbon units (Elovl2 and Elovl5) in relation to the carboxylic acid end of the FA [62].

**Figure 2:**
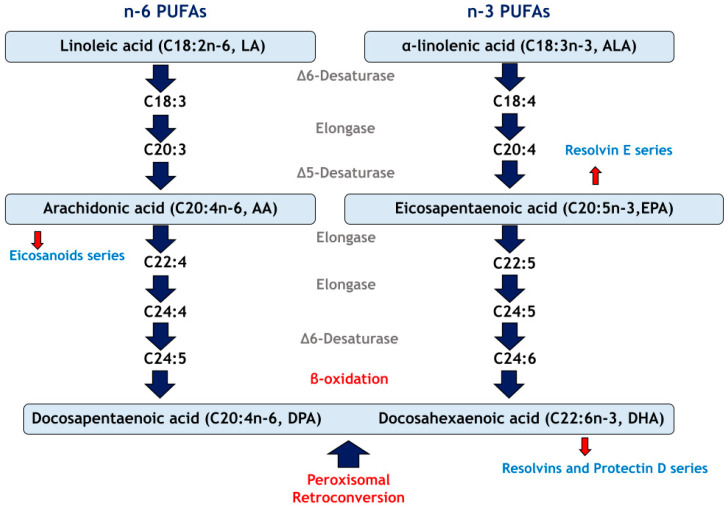
Metabolism of n-6 and n-3 PUFAs.

The main PUFAs derived from ALA are eicosapentaenoic acid (C20:5n-3, EPA) and docosahexaenoic acid (C22:6n-3, DHA), while arachidonic acid (C20:4n-6, AA) is the best-known LA derivative. DHA and AA are deposited in high concentrations in neuronal membranes and actively participate in brain and retinal development [60], and it is noteworthy that these FAs positively influence neurogenesis and neuronal differentiation [60]. Meanwhile, EPA regulates the metabolism of plasma lipids (especially triglycerides). The n-3 and n-6 PUFAs modulate the inflammatory response through the actions of their lipid mediators; for example, EPA and AA are precursors of eicosanoids (compounds of 20 carbon atoms), and DHA is the substrate for the production of docosanoids (derived from 22 carbon atoms) [61]. It is important to mention that lipid mediators derived from EPA and DHA have opposite effects to those derived from AA, thus contributing to resolving the inflammatory response [63]. The traditional dietary sources of LA are sunflower oil or corn, while AA is found in eggs, chicken or pork. ALA is found mainly in nuts (e.g., walnuts) and chia, flaxseed, canola, or soybean oils, and EPA and DHA are found mainly in fish such as tuna or salmon [60]. Figure 2 briefly shows the metabolism of n-6 and n-3 PUFAs.

## 6. PUFAs and the Gut Microbiome

Inflammatory cells usually contain high levels of n-6 and low levels of n-3 PUFAs [25]. The oral administration of these FAs can modify the EPA and DHA cell membrane contents and many pro-inflammatory cytokines (TNF-α, IL-1β, IL-6, and IL-8) and eicosanoids produced from AA metabolism are critical in the initial phase of inflammatory cascade activation [25]. Several studies that provided EPA and DHA supplements to healthy human volunteers have reported the decreased production of TNF-α, IL-1β, and IL-6 by LPS-stimulated monocytes or mononuclear cells [64,65,66]. The available studies show positive effects (decrease of cytokine production) when administered at 2 or more grams of EPA + DHA per day for six weeks [26]. In comparison, no effect was observed when less than 2 g of EPA + DHA per day was provided [26]. On the other hand, a reduction in pro-inflammatory cytokine production has been reported after n-3 PUFA supplementation for 6 weeks to 3 months [64,65]. In persons consuming a typical Western diet, phospholipids in the cells involved in the inflammatory response represent ~10–20% of AA, ~0.5–1% EPA, and ~2–4% DHA [67,68], with differences between the different phospholipid classes [69]. In rodents, dietary supplementation with fish oil providing n-3 PUFAs resulted in an increased content of these FAs in the cell membranes of lymphocytes [70], macrophages [71,72,73], and neutrophils [74,75]. The inflammatory response is part of a normal innate immune reaction as a controlled process of induction, regulation, and resolution of inflammatory events [76]. PUFAs can modulate the innate immunity and the course of chronic diseases like inflammatory bowel diseases [77]. For example, the administration of n-3 PUFAs in patients with Crohn’s disease significantly modified the cell membrane lipid profile in PBMC [78]. n-3 PUFAs compete against AA incorporation within membrane phospholipids, therefore replacing and blocking the production of pro-inflammatory eicosanoids, especially PG2 and LT4 [79]. EPA and DHA can reduce the cytokine-mediated induction of the inflammatory gene expression in chondrocyte cultures [80]. It has been proposed that the downregulation of inflammatory gene expression is mediated by nuclear factor kappa B (NF-κB) and peroxisome proliferator-activated receptors (PPAR) [81]. Of the transcription factors involved in inflammation, NF-κB is the most critical one involved in regulating inflammatory cytokines and adhesion molecules [82]. NF-κB is activated through a signaling cascade triggered via extracellular inflammatory stimuli. This process requires the phosphorylation of the NF-κB inhibitory subunit, which allows the translocation of the remaining NF-κB dimer to the nucleus [83]. Studies administering PUFAs show a reduction in the serum markers of inflammation, such as TNF-α and IL-6 [84]. Specifically, n-3 PUFAs have been shown to inhibit the phosphorylation and degradation of IκB proteins, which are necessary for the activation of NF-κB. By preventing this degradation, PUFAs reduce the translocation of NF-κB dimers (such as p65) to the nucleus, thereby limiting the expression of pro-inflammatory genes regulated by NF-κB [26,85].

## 7. n-3 PUFA: Effects on the Gut Microbiota

Recent studies have provided support for the idea that inflammation may be influenced by n-3 PUFAs, linking supplementation with EPA and DHA to the changes in the gut microbiota and dysbiosis. Dysbiosis is defined as an imbalance in the composition and function of the gut microbiota, characterized by reduced microbial diversity and alterations in the relative abundance of specific microbial groups. It has been observed that n-3 PUFAs can modulate the gut microbiota by influencing the type and abundance of the intestinal bacteria, altering the levels of inflammatory mediators such as endotoxins, and regulating the production of short-chain fatty acids (SCFAs) [86]. Several studies have found that n-3 PUFAs can reverse gut dysbiosis by increasing the abundance of Lactobacillus and Bifidobacterium and butyrate-producing bacteria. In line with this, Watson et al. [87] conducted a randomized trial evaluating the effect of n-3 PUFA supplements on the human gut microbiota of healthy adult subjects. They found an increase in the abundance of butyrate-producing bacteria, such as Bifidobacterium, Lachnospira, Roseburia, and Lactobacillus, along with an increase in the luminal SCFA levels. There is also evidence suggesting that n-3 PUFAs can reverse dysbiosis by restoring the Firmicutes/Bacteroides ratio and increasing the abundance of Lachnospiraceae, which promotes butyrate production [88].

In the context of intestinal inflammation, Zhao et al. [89] evaluated the effects of DHA-enriched phosphatidylserine in C57BL/6J mice exposed to bisphenol A. The results showed an increase in the relative abundance of beneficial bacteria, such as Akkermansia, Butyricicoccus, and Lachnospiraceae, which was associated with reduced intestinal permeability. This improvement in the intestinal barrier was also linked to the increased expression of tight junction proteins (claudin-1, occludin, and zonulin-1) and a reduction in inflammatory markers (IL-1β, IL-6, and TNF-α).

On the other hand, animal studies have demonstrated that n-3 PUFAs can increase the abundance of LPS-suppressing bacteria, such as Bifidobacterium, and reduce the abundance of LPS-producing bacteria, such as certain enterobacteria, thereby decreasing metabolic endotoxemia. This could improve the immune response by reducing the activation of inflammatory pathways induced by LPS, which act through the NF-kB pathway and the TLR4 receptor in intestinal epithelial cells [90].

**Figure 3:**
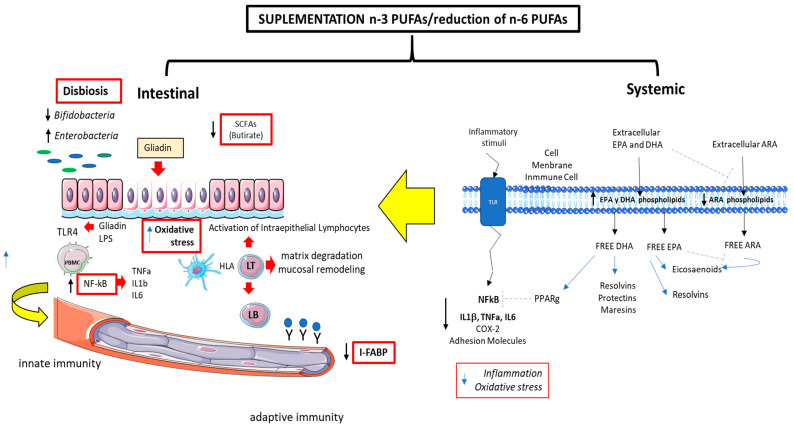
The anti-inflammatory role of n-3 PUFAS supplementation. n-3 PUFAs are potent anti-inflammatory molecules. Their intake promotes the enrichment of cellular membranes, facilitating anti-inflammatory signaling. Conversely, the involvement of omega-3 fatty acids in the pathophysiology of celiac disease and their potential impact on disease onset, progression, and treatment remains unexplored. Understanding their role in each of these processes is of interest.

It is important to note that the relationship between n-3 PUFAs and the gut microbiota is bidirectional, as it has been shown that the gut microbiota can indirectly modulate the absorption, bioavailability, and biotransformation of n-3 PUFAs, influencing their balance and function [29,91]. For example, Bifidobacterium has been described as a key genus in modulating the metabolism and uptake of n-3 PUFAs in the intestinal epithelium, which is associated with the increased levels of these fatty acids in the body [92]. However, further studies are needed to fully understand the specific mechanisms of this interaction. Additionally, some gut microorganisms have been reported to produce biologically active metabolites derived from n-3 and n-6 PUFAs. For example, Bacillus proteus and Lactobacillus plantarum are capable of converting the precursors, ALA and LA, into conjugated linoleic acid (CLA), a metabolite with well-established health benefits [93]. Figure 3

Given the evidence underscoring the documented beneficial effects of n-3 polyunsaturated fatty acids (PUFAs) on gut health—especially in resolving inflammatory processes (like Crohn’s disease) and modulating the gut microbiota—we think it worth investigating the relationship between n-3 PUFA supplementation, the composition and diversity of the gut microbiota, short-chain fatty acid (SCFA) levels (both fecal and systemic), and intestinal inflammation in celiac patients.

## 8. Fatty Acids and Celiac Disease: Is There a Causal Relationship?

When celiac disease (CD) is triggered, the most prominent event in the intestinal mucosa that leads to the clinical manifestation of the disease is the inflammatory process. Consequently, managing inflammation emerges as a promising approach to improving the disease management in CD. However, the potential relationship between inflammation, PUFA status in the blood, and the clinical response remains unclear, with data still being limited and inconclusive [94,95]. One notable study conducted in a cohort of infants at high risk of CD followed for 8 years revealed that those who later developed CD had a dramatically different phospholipid profile. This altered profile was detected before the appearance of typical CD serum autoantibodies, distinguishing infants who would eventually develop the disease from those who would not, even before symptom appearance [96].

In comparison to healthy controls, a general reduction in the proportion of n-3 PUFAs has been documented in patients with celiac disease. This reduction was notably more pronounced in patients with active CD compared to those adhering to a gluten-free diet (GFD) [97]. Additionally, a study that focused on AA-derived mediators, such as 15-hydroxyeicosatetraenoic acid (15-HETE), found elevated levels in newly diagnosed patients with CD [98]. Another key study assessed the fatty acid profiles in erythrocytes from newly diagnosed CD patients and reported significant alterations, including a 2.08 times higher concentration of arachidonic acid (AA) and a 1.4 times higher AA/DHA ratio [99]. Moreover, research has shown that intestinal epithelial cells release AA when stimulated by gliadin, a key component of gluten, thereby contributing to the inflammatory status observed in CD. Interestingly, DHA has been shown to inhibit this release of AA, suggesting a potential protective role of DHA against inflammation in CD [100].

Despite these insightful findings, the available data on the relationship between fatty acid intake and inflammation in CD patients are scarce, and further studies are necessary to draw definitive conclusions. To better understand these complex interactions, we compiled a comprehensive table analyzing all the existing studies on fatty acids and CD, offering a clearer picture of the current state of research. This table (Table 1) serves to highlight both the promising insights and the gaps that need to be addressed in future investigations. Further research is crucial for establishing whether specific dietary interventions targeting fatty acids may be an effective strategy to modulate inflammation and improve the clinical outcomes in patients with celiac disease. In Summary Table 1, the studies that have used n-3 in patients with celiac disease are shown.

## 9. Therapeutic Application of n-3 PUFAs

There is no solid data on CD, but in the therapeutic context, the oral route is the most common route used to supplement n-3 fatty acids. This includes the intake of concentrated n-3 PUFA supplements, such as fish oil or flaxseed oil, or the direct incorporation of dietary sources rich in these fatty acids [26]. The choice of the administration method depends on factors such as the clinical condition, intestinal absorption, and the individual response to treatment [88]. The dosage of n-3 fatty acids varies depending on the disease and the patient’s condition. In inflammatory bowel diseases (IBDs), such as ulcerative colitis and Crohn’s disease, doses between 1000 and 3000 mg per day of EPA and DHA have been shown to have beneficial anti-inflammatory effects [103]. However, the specific dosage can be adjusted based on the severity of the disease and the individual clinical response. In conditions such as irritable bowel syndrome (IBS), the dosage range may differ, and administration should be carefully monitored to optimize the results and avoid adverse effects. Furthermore, the balance between n-3 and n-6 fatty acids in the diet is crucial to maximize the therapeutic outcomes [104].

## 10. Conclusions

Celiac disease (CD) is a systemic autoimmune disorder that continues to impose significant health and economic challenges globally. The cornerstone of CD treatment is the adherence to a gluten-free diet, but the effectiveness of this approach varies widely among patients, highlighting the need for alternative long-term management strategies. The clinical manifestations of CD are primarily triggered by inflammatory processes in the small intestinal mucosa, which are influenced by genetic factors and gluten exposure, ultimately impacting various body systems. This inflammatory response involves multiple contributing factors. While research indicates that n-3 PUFAs exhibit anti-inflammatory properties in conditions like Crohn’s disease, there is currently no strong evidence to suggest similar effects in CD. Additionally, n-3 PUFAs have been shown to influence the composition and diversity of the gut microbiota, as well as intestinal inflammation. The diet can positively affect the microbiota, especially when promoting butyric acid-producing bacteria, which can improve dysbiosis and the immune response. The reviewed data indicate that supplementing celiac patients with oral n-3 PUFAs may help modify the immune response dysbiosis and contribute to the management of CD.

## Figures and Tables

**Table 1:** Studies on celiac disease and the use of n-3 polyunsaturated fatty acids.

Study	Patients	Age Group	Location/Sampling Methods	Outcomes	**Main Findings**
Steel et al. (2006) [95]	Patients with active CD (n = 7) Patients with CD under treatment (n = 6) Controls (n = 11)	Active CD: Mean 75 months (range = 12–174 months). CD under treatment: Mean 98.5 months (range = 43–195 months). Controls: Mean 34 months (range = 18–176 months).	Duodenal biopsies and blood samples were obtained after an overnight fast.	Serum from patients with active CD: Increased levels of palmitoleic acid (16:1n-7), oleic acid (18:1n-9), and Mead acid (20:3n-9); decreased levels of long-chain saturated fatty acids such as behenic acid (22:0) and lignoceric acid (24:0). Serum from patients with CD under treatment: No significant differences from controls, except for a higher ratio of arachidonic acid (AA; 20:4n-6) to docosahexaenoic acid (DHA; 22:6n-3). Intestinal mucosa from patients with active CD: Elevated l n-6 PUFA levels, decreased linoleic acid, increased AA, and a 20:3n-9/20:4n-6 ratio, indicating a possible deficiency of essential fatty acids.	Active CD: The fatty acid profile in the intestinal mucosa shows an imbalance, particularly in the n-6 series, with an increased conversion of n-6 fatty acids to their derivatives. Serum levels do not accurately reflect the fatty acid status in the intestinal mucosa, indicating that tissues may vary in their ability to retain fatty acid status during essential fatty acid deficiency. CD under treatment: The fatty acid profiles in serum and intestinal mucosa did not show significant differences compared to controls, except for a higher ratio of AA/DHA.
Bai et al. (2024) [101]	13,403 subjects who responded to the National Health and Nutrition Examination Survey (NHANES) 2009–2014. Of these, 48 cases of CD were identified.	Adults aged 20 years or older (50.28 ± 17.34)	The data come from the National Health and Nutrition Examination Survey (NHANES) in the U.S. (2009–2014). A representative sample of non-institutionalized adults in the U.S. was used, with dietary intake and health information collected through interviews and questionnaires.	Cross-sectional study: No significant association was found between the dietary intake of n-3 PUFAs and CD. Mendelian randomization: A significant correlation was found between serum levels of n-3 PUFAs and CD.	The dietary intake of n-3 PUFAs did not show a significant association with CD in the cross-sectional analysis. However, Mendelian randomization revealed a positive causal relationship between the serum levels of n-3 PUFAs and CD, suggesting that the elevated blood levels of n-3 PUFAs may be associated with an increased risk of CD.
Tárnok et al. (2015) [102]	Patients with utreated CD (n = 28), patients with CD and diabetes mellitus (CDDM) (n = 8), and healthy controls (n = 21)	The mean age was 13.1 years for patients with untreated CD, 11.6 years for patients with CD and diabetes mellitus, and 13.3 years for control individuals.	Plasma sample. Comparison of fatty acid composition using high-resolution gas–liquid chromatographic analysis.	Significantly reduced levels of docosapentaenoic acid (C22:5n-3), docosahexaenoic acid (C22:6n-3), and total n-3 (n-3 PUFA) were found in the chronic diabetes mellitus (CDDM) group compared to the controls and patients with CD.	Children with chronic diabetes mellitus (CDDM) exhibited the reduced availability of n-3 PUFA and long-chain n-3 PUFA in circulating lipids. Diabetes mellitus (DM) has a significant impact on the composition of plasma fatty acids in children, while CD alone did not show significant alterations in the metabolism of PUFAs in the plasma.
Vincentini et al. (2011) [100]	Caco-2 cells, an intestinal epithelial cell line derived from a human colon adenocarcinoma, were used as in vitro models of CD.	—	Caco-2 cells were exposed to gliadin peptides (PT-gls) and docosahexaenoic acid (DHA) under various conditions. The exposure lasted for up to 24 h	Exposure to PT-gl increased the release of AA, the expression of cyclooxygenase-2, the activity of cytosolic phospholipase A2 (cPLA2), and the release of prostaglandin E2 and interleukin-8 in the culture medium. Simultaneous exposure to DHA and PT-gl prevented these increases.	Intestinal epithelial cells (Caco-2) release AA in response to gliadin exposure, which contributes to celiac inflammation. DHA may modulate celiac inflammation by inhibiting the release of AA by intestinal epithelial cells.
Solakivi et al. (2009) [97]	Patients with CD (n = 50) and healthy controls (n = 61)	The mean age in CD patients was 44 years (range of 16–71 years), and the mean age of the controls was 40 years (range of 20–58 years).	Blood samples (serum) were taken after an overnight fast. Serum fatty acids were analyzed using capillary gas chromatography.	In patients with active CD, saturated and monounsaturated fatty acids were elevated, while polyunsaturated fatty acids were reduced compared to controls. After one year on a GFD, polyunsaturated fatty acids increased but remained low compared to controls.	In patients with active CD, essential fatty acid deficiency was reflected in decreased polyunsaturated fatty acids and increased saturated and monounsaturated fatty acids. Although the one-year GFD showed clinical and serological improvement, the fatty acid profiles remained abnormal, and a high triene/tetraene ratio was observed, which may predispose individuals to dermatitis and neurological disorders.
Riezzo et al. (2014) [99]	Patients with CD (n = 20), healthy subjects (controls) (n = 20)	Subjects with CD, mean age: 34.0 years (±1.7 years) Control subjects, mean age: 40.2 years (±2.5 years)	Erythrocyte samples from newly diagnosed CD patients and healthy subjects. The comparison was made at the time of diagnosis and after 1 year of intervention with a GFD.	Patients with CD showed AA levels 2.08 times higher than those in healthy subjects. Additionally, the ratio of AA to dihomo-γ-linolenic acid was significantly lower (2.01 times) in CD patients, while the ratio of AA to docosahexaenoic acid was 1.40 times higher. After one year on a GFD, the fatty acid concentrations in patients with CD still differed from those observed in healthy subjects.	AA could be considered a potential marker for CD. Patients with CD have an inefficient synthesis of polyunsaturated fatty acids from their precursors. The intervention with a GFD is not sufficient to fully restore the fatty acid concentrations to their normal levels. Lipid analysis of erythrocytes may be a useful and less invasive method compared to evaluating the fatty acid pattern in the intestinal mucosa for assessing therapeutic interventions in patients with CD.
Van Hees et al. (2014) [94]	Patients with CD (n = 71), 65% of whom had one or more current psychiatric diagnoses, primarily anxiety disorders. Healthy controls (n = 31).	Patients with CD: 18–93 years, mean age 54 years. Healthy controls: 22–66 years, mean age 51 years.	Patients with CD were recruited from the Dutch Celiac Association, and healthy controls were from the population-based “Normquest” study. Dietary intake was assessed using a 203-item food frequency questionnaire, and the serum levels of EPA and DHA were compared using analysis of covariance. Serum PUFA levels were determined via gas chromatography.	Dietary intake: No significant differences in EPA and DHA intake between patients and controls. Serum levels: DHA was significantly higher in patients with CD (1.72%) compared to controls (1.28%). EPA showed no significant differences.	Patients with CD have higher serum levels of DHA compared to controls, but this is not due to higher dietary intake. There is no significant association between EPA/DHA levels and major depressive disorder (MDD) in patients with CD. Adherence to a GFD and the duration of the diet do not significantly affect the serum DHA levels or DHA intake.
